# Stroke with atrial fibrillation or atrial flutter: a descriptive population-based study from the Brest stroke registry

**DOI:** 10.1186/s12877-015-0067-3

**Published:** 2015-06-11

**Authors:** Virginie Jannou, Serge Timsit, Emmanuel Nowak, François Rouhart, Philippe Goas, François-Mathias Merrien, Irina Viakhireva-Dovganyuk, Anne Tirel-Badets, Armelle Gentric

**Affiliations:** Department of Internal Medicine and Geriatrics, University Hospital of Brest, Boulevard Tanguy Prigent, 29200 Brest, Bretagne France; Neurology and Stroke Department, University Hospital of Brest, Boulevard Tanguy Prigent, 29200 Brest, Bretagne France; University Hospital of Brest, CIC INSERM, Boulevard Tanguy Prigent, 29200 Brest, Bretagne France

**Keywords:** Stroke, Atrial fibrillation, CHADS2, Anticoagulation, Registry

## Abstract

**Background:**

In the 1990s, epidemiological studies estimated the prevalence of stroke caused by atrial fibrillation (AF) at about 15 %. Given the aging population, there is a rise in the number of AF patients. AF prevention guidelines based on clinical practice and the literature have been published and updated since 2001. Implementation seems to have an impact on the prescription of vitamin K antagonist (VKA). During the last 20 years, few population-based studies have focused on the prevalence of atrial arrhythmia (AA) in patients with stroke. The objective of the present prospective study, using data from 2008, was to evaluate the prevalence of AA (atrial fibrillation/flutter) in patients with stroke and the impact of implementing AF guidelines.

**Methods:**

The prevalence of AA was studied in patients diagnosed with stroke from January 1 to December 31, 2008 in the population-based Stroke Registry of Brest, France (total population, 363,760 according to the 2008 census, with 295,553 aged 15 years or older). Guidelines implementation was assessed in terms of antithrombotic therapy (VKA, antiplatelet agent, none), and the CHADS2 (Congestive heart failure, Hypertension, Age > 75 years, Diabetes mellitus, and prior Stroke or transient ischemic attack).

**Results:**

851 cases of stroke were identified. The prevalence of AA was 31.7 % (*n* = 264), and increased with age from < 20 % in patients aged 45 to 54 years to nearly 50 % in patients ≥ 85 years. In patients with AA, 231 strokes were ischemic, 28 hemorrhagic and 5 undetermined. At time of stroke, AA was known in 207 patients (78.4 %). 54 of the 152 patients with CHADS2 score ≥ 2 (35.5 %) were treated with VKA; this proportion decreased with age: 50 % between 50 and 74 years, 43.8 % between 75 and 84 years, and 25 % at 85 years and older.

**Conclusion:**

The prevalence of AA in the population-based Brest Stroke Registry in 2008 was higher than that reported by studies conducted 20 years ago. Despite publication of AF prevention guidelines, VKA prescription and use in elderly patients were significantly low.

## Background

Atrial fibrillation (AF) is a public health problem because of its epidemiology and severity: it is the most common form of cardiac arrhythmia, affecting approximately 1 % of adults [1]. Prevalence increases with age, from < 0.5 % at 40 to 50 years, to > 10 % at 80 years or older [1]. In population-based studies, incidence increased from < 0.1 % per year before 40 years to > 1.5 % after 80 years [2]. Given the aging population, the number of patients with AF is expected to rise. AF is associated with increased risks of: mortality [3], heart failure [4], dementia [5] and systemic embolism, with a 5-fold increase in risk of stroke [6]. Stroke due to AF shows higher mortality and worse outcome than stroke due to atherosclerosis [7]. AF is commonly associated with other risk factors for stroke: age, female gender, hypertension, prior stroke or transient ischemic attack (TIA), systolic congestive heart failure, diabetes mellitus, and vascular disease [8]. Warfarin has been shown to be highly effective in preventing stroke in AF [9, 10]. American (ACC/AHA) and European (ESC) guidelines have been established for clinical practice. The risk of thromboembolism (TE) was evaluated on the basis of CHADS2 score (Congestive heart failure, Hypertension, Age > 75 years, Diabetes mellitus, and prior Stroke or transient ischemic attack) scores in 2001 [11] and 2006 [12]; the CHADS2 score was replaced by CHA2DS2 Vasc in 2010 [13]. Thromboprophylaxis in AF requires assessment of both stroke and bleeding risks. Despite its proven benefit, warfarin is underused in AF patients, and in particular in the elderly [14–16]. TE risk in atrial flutter has been less well assessed than in AF, but is estimated to be intermediate compared to sinus rhythm or AF [17].

In the 1990s, before the publication of AF prevention guidelines, epidemiological studies estimated prevalence of AF-related stroke at about 15 % [18].

During the last 20 years, few population-based studies have focused on the prevalence of arrhythmia in stroke. Given the aging population, there is a rise in the number of AF patients. AF prevention guidelines based on clinical practice and the literature have been published and updated since 2001. Implementation seems to have an impact on the prescription of vitamin K antagonist (VKA). The main objective of the present prospective study based on 2008 data was to evaluate the prevalence of atrial arrhythmia (AA) (atrial fibrillation/flutter) and the impact of implementation of prevention guidelines on CHADS2 scores in a stroke population. The secondary objective was to calculate the CHA2DS2Vasc score to estimate the number of patients at high risk of TE and requiring anticoagulation according to current guidelines.

## Methods

The prevalence of AA was studied in patients diagnosed with stroke from January 1, 2008 to December 31, 2008, in the population-based Stroke Registry of Brest (France).

### Study population

A description of the Brest Stroke Registry has been published previously [19].

The Brest Stroke Registry is a population-based registry covering the Brest area, in northwest France: population, 363,760 according to the 2008 census, with 295,553 aged 15 years or older.

### Case ascertainment

All residents of the Brest area with stroke were taken into account. To ensure exhaustive ascertainment, data were collected from several local sources: two teaching hospitals (Brest University Hospital and Clermont-Tonnerre Military Hospital, both in Brest) and one general hospital (Ferdinand Grall Hospital, in nearby Landerneau), general practitioners, three neurologists in private practice, private radiology centers, nursing homes, and finally from death certificates, providing data for fatal stroke in non-hospitalized subjects.

### Stroke definition and classification

Two previously described definitions of stroke were considered [19]: 1) new focal neurological deficit with symptoms and signs in line with the World Health Organization definition of stroke [20], lasting for more than 24 h (patients with focal neurological deficit who died within the first 24 h were also included); and 2) all neurological focal deficits lasting at least 1 h or resolving within 1 h but with abnormal brain imaging associated with a clinically relevant picture [19]. An abnormal image was defined as an image visible on CT or MRI scan showing ischemic or hemorrhagic stroke, the location of which may explain the clinical presentation. Subarachnoid hemorrhage was excluded from the study. Subtype diagnosis by neurologists of the Department of Neurology was based on clinical examination, cerebral imaging (CT or MRI), and complementary investigations including carotid and vertebral ultrasonography, and echocardiography. Stroke was classified as ischemic, hemorrhagic or “unknown” subtype according to the Oxfordshire Community Stroke Project (OCSP) classification [21]. Cerebral infarctions were classified according to the Trial of Org 10172 in Acute Stroke Treatment classification (TOAST) [22] and the Stop Stroke Study TOAST (SSS-TOAST) [23]: thrombosis or embolism due to atherosclerosis of a large artery, embolism of cardiac origin, occlusion of a small blood vessel, other determined cause, or undetermined cause (two or more causes identified, negative assessment, or incomplete assessment). An automated version of the SSS-TOAST was used for the registry [24].

AA was diagnosed in patients with atrial fibrillation/flutter known at the time of stroke, and/or in patients classified as having permanent or paroxysmal atrial fibrillation or atrial flutter on the basis of the SSS-TOAST for cerebral infarction. In patients with intracranial hemorrhage and “undetermined” stroke with AA unknown at the time of the stroke, all electrocardiograms (ECG) were analyzed and the general practitioner was contacted by phone to check for the history of AA.

In patients with AA known at the time of the stroke, the CHADS2 score was calculated to assess the impact of implementation of TE prevention guidelines. (CHADS2, and not the later CHA2DS2 Vasc, was the score in use at the time of the study [12]). The following data were recorded: age according to demographic data; history of hypertension, diabetes mellitus, and prior stroke or TIA according to medical records, and history of congestive heart failure on SSS-TOAST for cerebral infarction. The CHADS2 score was obtained by assigning and adding points: 1 point for each moderate risk factor (heart failure, hypertension, age, and diabetes mellitus) and 2 points for history of stroke or TIA. Patients were assigned to one of three classes of TE-related stroke risk: low risk (CHADS2 score = 0), intermediate risk (CHADS2 score = 1), or high risk (CHADS2 score ≥ 2). Patients with missing data were classified as “unknown”. For each patient for whom CHADS2 was calculated, the antithrombotic therapy prescribed at the time of stroke was classified as follows: no therapy, platelet aggregation inhibitors (aspirin and/or clopidogrel), or anticoagulation with VKA. The International Normalized Ratio (INR) was measured at admission. The prescribed antithrombotic therapy was compared to the recommended treatment according to CHADS2 score: i.e., VKA at a dose adjusted to achieve an INR within the therapeutic range (between 2 and 3) in high-risk patients (CHADS2 score ≥ 2); aspirin at a dose of 75 to 325 mg daily in low risk patients (CHADS2 score = 0); or VKA or aspirin according to the clinical context and patient’s preference in patients at moderate risk of TE (CHADS2 score = 1).

The CHA2DS2 Vasc score was then calculated for patients with AA known at the time of stroke, according to the scoring used for CHADS2. Additional data were collected: female gender from demographic data, peripheral artery disease and prior myocardial infarction from medical records, and aortic plaque (>4 mm) according to SSS-TOAST for cerebral infarction. Patient distributions according to CHADS2 and CHA2DS2 Vasc scores were compared.

### Statistical analysis

Statistical analysis used SAS software. EpiData 3.1 software was used for registry data entry. Median and mean ages were calculated, with quartiles and standard deviations respectively. Frequencies were calculated for gender, prior stroke, prior TIA, myocardial infarction, hypertension, and diabetes mellitus. Missing data were also calculated and variables with more than 10 % missing data were discarded. Prevalence of AA according to gender and age was calculated.

### Ethics

The Brest Stroke Registry was approved by the national ethics committee (CNIL) and the French Public Health Watch Institute (InVS).

## Results

In 2008, 851 strokes were recorded: 733 ischemic (86 %), 96 hemorrhagic (11 %), and 22 undetermined (3 %). 383 of the patients were male (45 %) and 468 female. Mean age was 75.5 ± 13.6 years and median age 79 years. Sixteen patients were excluded because they did not undergo ECG or cardiac monitoring at admission, medical history was unknown or the general practitioner could not provide data on possible AA (general practitioner unknown, cessation of private practice, or archiving of medical records).

**Fig. 1:**
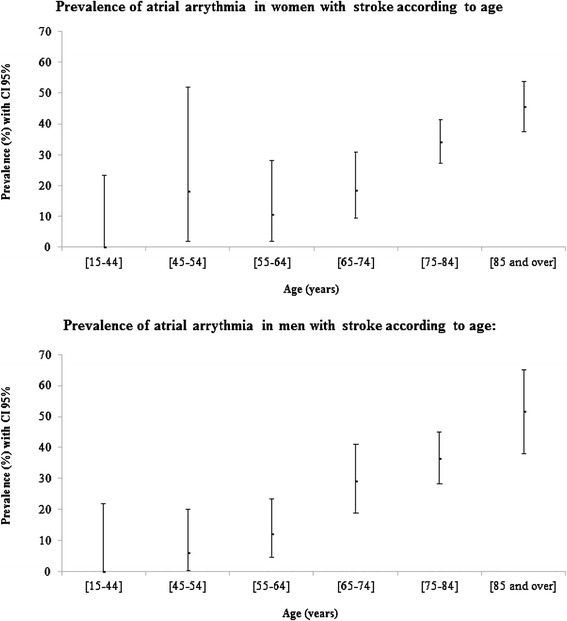
Prevalence of atrial arrhythmia-related stroke in 2008, by age and gender. Legend: CI: Confidence Interval

**Fig. 2:**
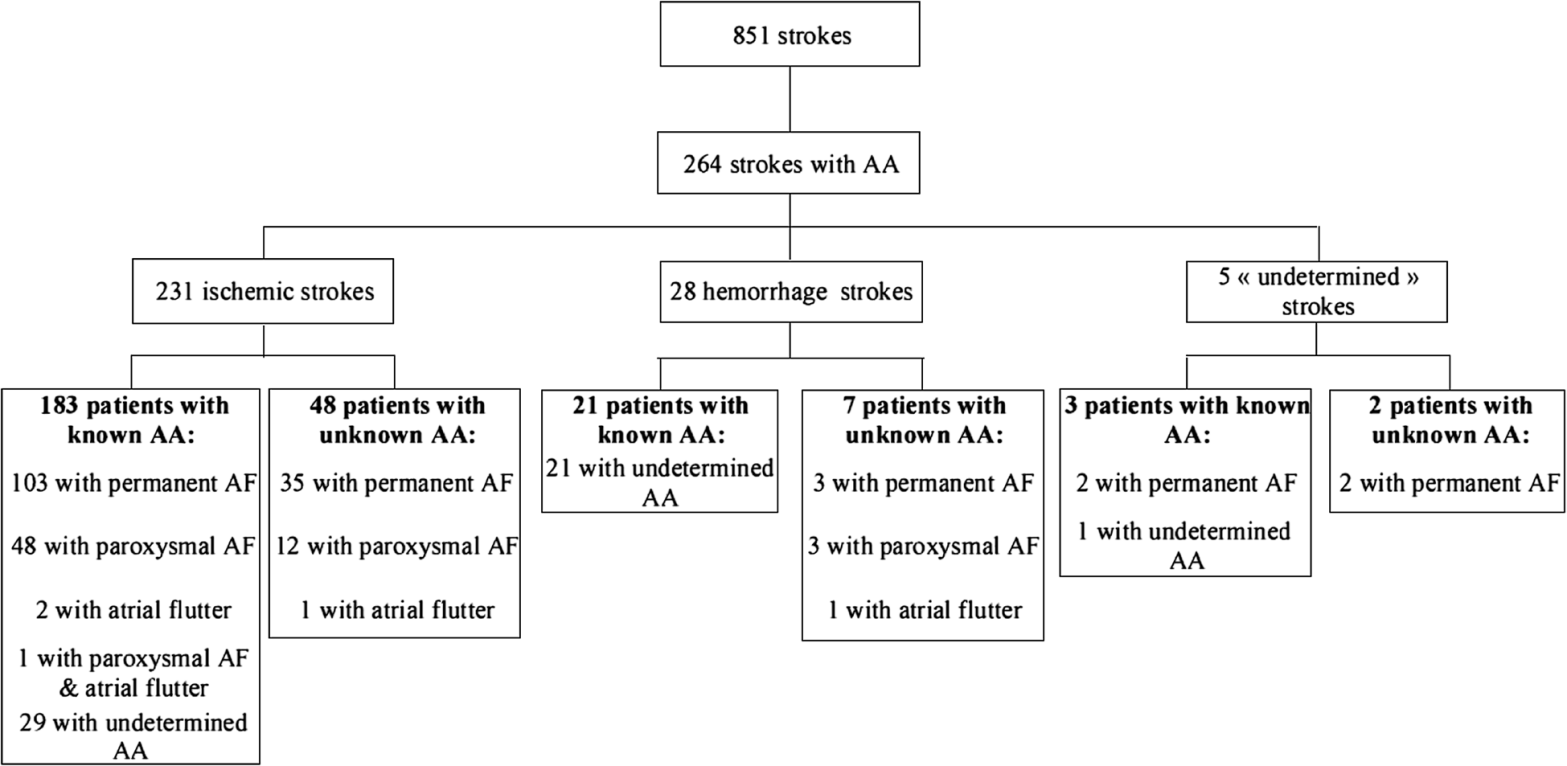
Flowchart of patients throughout the study. Legend: AA: Atrial Arrhythmia; AF: Atrial Fibrillation

264 of the remaining 835 patients (31.7 %) had AA (AF or flutter). Mean and median ages for patients with AA were 81.1 ± 8.9 years and 83 years (range, 77–87 years), respectively. There were 152 women (57.6 %) and 112 men (42.4 %). As shown in Fig. 1, prevalence of AA increased with age in both genders. 231 of the 264 strokes associated with AA were ischemic (87.5 %), 28 hemorrhagic (10.6 %), and 5 undetermined (1.9 %). The distribution between atrial fibrillation and atrial flutter is described in Fig. 2: 138 patients with ischemic stroke had permanent AF (59.7 %), 61 paroxysmal AF (26.4 %) and 3 atrial flutter (1.3 %); AA type was undetermined in 29 (12.6 %). In 207 patients (78.4 %), AA was already known at the time of stroke. The demographic and baseline characteristics of the 264 patients are presented in Table 1.

**Table 1:** Demographics and Baseline Characteristics of the Population

	Number N (%)	Missing data N (%)
Median age (quartiles)	83 (77–87)	0
Mean age and SD	81.1 +/−8.9	0
Male	112 (42.4 %)	0
Female	152 (57.6 %)	0
Atrial arrhythmia	207 (78.4 %)	3 (1.1 %)
Prior stroke	68 (25.8 %)	8 (3 %)
Prior TIA	30 (11.4 %)	17 (6.4 %)
Myocardial infarction	43 (16.4 %)	20 (7.6 %)
Hypertension	175 (66.3 %)	11 (4.2 %)
Diabetes mellitus	37 (14 %)	14 (5.3 %)

Legend: SD: Standard deviation, TIA: Transient Ischemic Attack

231 of the 733 patients with ischemic stroke recorded in 2008 had AA (31.5 %).

CHADS2 score was calculated for the 207 patients with AA known prior to stroke: 10 (4.8 %) scored 0 and 33 (15.9 %) scored 1. Antithrombotic therapy according to CHADS2 score is shown in Table 2. 54 of the 152 patients (73.4 %) with CHADS2 score ≥ 2 received VKA (35.5 %), associated with aspirin in 3 cases. INR (<2, [2, 3], >3) according to CHADS2 score in patients on VKA is shown in Table 3; in 2 cases, INR was undetermined.

**Table 2:** Antithrombotic therapy according to CHADS2 score

	aspirin	clopidogrel	aspirin + clopidogrel	VKA	no antithrombotic therapy
CHADS2 unknown	1	0	0	8	3
CHADS2 = 0	2	2	0	2	4
CHADS2 = 1	7	0	0	12	14
CHADS2 ≥ 2	60	8	1	54, including 3 patients on both aspirin and VKA.	29

Legend: VKA: Vitamin K Antagonist

**Table 3:** INR according to the CHADS2 score, in patients with VKA

	INR < 2	2–3	INR > 3
CHADS2 = 0	2 (100 %)	0	0
CHADS2 = 1	9 (75 %)	3 (25 %)	0
CHADS2 ≥ 2	29 (55.8 %)	18 (34.6 %)	5 (9.6 %)

Legend: INR: International Normalized Ratio, VKA: Vitamin K Antagonist

The proportion of patients at high risk of TE with CHADS2 score ≥ 2 receiving VKA decreased with age: 50 % for 50–74 years, 43.8 % for 75–84 years, and 25 % for ≥ 85 years.

**Fig. 3:**
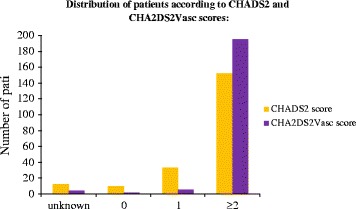
Distribution of patients with atrial arrhythmia known prior to stroke according to CHADS2 and CHA2DS2 scores

CHA2DS2 Vasc was also calculated in the 207 patients with AA known prior to stroke. Distribution according to CHADS2 and CHA2DS2 Vasc scores is shown in Fig. 3: 195 patients were classified as being at high risk of TE on CHA2DS2Vasc, compared to 152 on CHADS2.

Nine of the 28 patients (32.1 %) with hemorrhagic stroke and AA were on antiplatelet therapy, 14 (50 %) on VKA, 1 on antiplatelets and VKA, and 4 (14.3 %) had no antithrombotic therapy. For the 15 patients on VKA, INR was within therapeutic range, > 3 and <2, in 10, 2 and 3 patients, respectively.

## Discussion

The prevalence of AA-related stroke was 31.7 % in our registry, higher than in previous population-based studies, where proportions of AF-related stroke were between 18 and 24 % [25, 26]. These population based-studies were conducted before the publication of the AF guidelines.

Given the differences in age and gender distributions in AA-associated stroke, comparison of AA prevalence is difficult. However, the present results are consistent with those reported by the North Dublin Population Stroke Study in 2010 [27]. In this recent study, including patients between 2005 and 2006, the authors observed that 31.2 % of strokes (ischemic or hemorrhagic) were associated with AF. We compared our results with those of Marini et al.[26] and the North Dublin Population Stroke Study [27] on prevalence analysis by age group and gender: our results showed a higher prevalence than Marini et al. in men for all age groups and for women < 60 years and women > 80 years. Prevalence analysis by age group showed lower prevalence for patients < 84 years than in Dublin registry, but slightly higher prevalence for patients aged ≥ 85 years.

Thus the discrepancy between the present AA-related stroke prevalence and those reported before the AF guidelines may be explained by the difference in AA diagnosis technology and patient management. In 2008, electrocardiograms were monitored in day-care, whereas they were not routinely performed 20 years ago (e.g., in the 1992 Oxfordshire study [18]). Since then, the widespread use of Holter ECG combined with the development of stroke units and telemetry monitoring during the first 48 h of acute ischemic stroke have led to higher rates of diagnosis of paroxysmal AF not previously diagnosed by medical history, ECG or 24 h Holter ECG. The present study thus included patients with paroxysmal AF or atrial flutter, whereas previous studies included only patients with permanent AF. In our registry, 61 ischemic strokes were associated with paroxysmal AF (26.4 %), and 4 with atrial flutter only (1.5 %). The increasing prevalence of stroke associated with AA could partly be explained by population aging in developed countries [28]. In the present study, prevalence was also higher than in hospital-based studies [29], probably because our population included a higher proportion of very elderly patients, at high risk of AF.

In the present study, AA was known prior to stroke in almost 80 % of cases, probably reflecting good AA diagnosis. In the North Dublin Population Stroke Study [27], 54.4 % of patients had diagnosis of AF prior to stroke: in other studies, AF was known at the time of stroke in 65 % to 83 % of patients [14, 30, 31].

36.7 % of our patients with AA diagnosed prior to stroke were on VKA, comparably to other hospital- or population-based studies [15, 27, 31, 32]. This under-prescription of oral anticoagulants has also been observed in the general population (out- and in-patients), despite the absence of known contraindications [33].

Furthermore, in our registry, only 35.5 % of the high TE risk patients with CHADS2 score ≥ 2 were treated with VKA as recommended, and 45.4 % were treated with antiplatelet agents. Underuse of VKA therapy in the present study increased with age: only 25 % of patients aged ≥ 85 years were on VKA. This underuse of anticoagulant therapy in the elderly was observed in previous studies: < 60 % of patients in the report by Ogilvie et al. [14]. Several hypotheses may explain this: physician’s underestimation of AF-related TE risk; physician’s previous bad experience of bleeding, especially in patients with cognitive dysfunction [34] or at risk of falls [35]; underestimation of the bleeding risk associated with aspirin [36]; and difficulty in achieving or maintaining target INR. At the time of stroke, INR was within therapeutic range in only 36 of the patients treated with VKA (44.4 %). Several clinical studies have shown that INR remains within therapeutic range only 29 % to 75 % of the time [37]. Genetic factors, chronic comorbidity, diet, concomitant medication and patient compliance can have an impact on target INR management [38].

At the start of the present study, CHADS2 scores were calculated in compliance with the 2006 AF prevention guidelines. The more recent CHA2DS2 Vasc score revealed a larger proportion of patients requiring anticoagulation, highlighting the importance of updating and implementing guidelines.

AA prevalence was assessed in all types of stroke (ischemic, hemorrhagic and “undetermined”) for two reasons. Firstly, patients with “undetermined” stroke a) died before either arriving at hospital or undergoing brain imaging, and b) were either living at home or institutionalized with serious comorbidities. “Undetermined” stroke included severe and fatal stroke. It is known that stroke associated with AF is more serious than stroke without AF, due to a higher 30-day case-fatality rate [26]. This led us to include patients with “undetermined” stroke. Secondly, hemorrhagic stroke was included in order to estimate the proportion of hemorrhagic strokes associated with AA among strokes in general, and to determine the impact of oral anticoagulants on these patients.

The major strength of the present study was its design: a population-based study including both hospitalized and non-hospitalized patients. The study evaluated an older population than in hospital-based studies. Case ascertainment was quite exhaustive, based on multiple sources of information. The high level of completeness and reliability of the Brest Stroke Registry was established in our previous study [19]. The present results represent the step after preliminary validation of the Registry in 2014.

The limitation of the study was that, given the lack of adequate duration of AA monitoring in early 2008 (i.e., before the opening of specialized stroke units), some cases of paroxysmal AA may have gone undiagnosed.

## Conclusion

The prevalence of AF-related stroke is high in the elderly and, despite prevention guidelines published 20 years ago, only a third of the patients at high TE risk are treated with VKA. We believe that today, with the advent of novel oral anticoagulants accompanied by the more recent AF guidelines [39] based on CHA2DS2 Vasc score, and extensive patient education programs, physicians are more alert in the management of patients with AA.

## Abbreviations

AA: Atrial arrhythmia
VKA: Vitamin K antagonist
AF: Atrial fibrillation
TIA: Transient ischemic attack
TE: Thromboembolism
MRI: Magnetic resonance imaging
OCSP: Oxfordshire Community Stroke Project
TOAST: Trial of Org 10172 in Acute Stroke Treatment
SSS TOAST: Stop Stroke Study TOAST
ECG: Electrocardiograms
INR: International Normalized Ratio
CNIL: Commission Nationale de l’Informatique et des Libertés
InVs: Institut de Veille Sanitaire
